# Milestones in Tremor Research: 10 Years Later

**DOI:** 10.1002/mdc3.13418

**Published:** 2022-02-26

**Authors:** Roberto Erro, Alfonso Fasano, Paolo Barone, Kailash P. Bhatia

**Affiliations:** ^1^ Department of Medicine, Surgery and Dentistry “Scuola Medica Salernitana”, Neuroscience Section University of Salerno Baronissi Italy; ^2^ Edmond J. Safra Program in Parkinson's Disease Morton and Gloria Shulman Movement Disorders Clinic, Toronto Western Hospital, UHN Toronto Ontario Canada; ^3^ Division of Neurology University of Toronto Toronto Ontario Canada; ^4^ Krembil Brain Institute Toronto Ontario Canada; ^5^ Department of Clinical and Movement Neurosciences University College London London United Kingdom

**Keywords:** essential tremor, dystonic tremor, Parkinson's disease, sensors, MRgFUS

## Abstract

Major progress has occurred during the last decade in the field of tremor. From the clinical standpoint, a new classification has completely revised the nosology of tremor syndromes and has re‐conceptualized essential tremor as a syndrome rather than a single disease entity, fueling an ongoing enlightened debate. Significant advances have been obtained in terms of instrumental measurement of tremor, remarking on the possibility of developing novel treatment strategies based on tremor characteristics, namely tremor‐phase. Moreover, a better understanding of the pathophysiological mechanisms has further led to the suggestion of refining the classification of tremor syndromes according to their driving underpinnings. Finally, surgical options such as deep brain stimulation and focused ultrasound thalamotomy are now part of the therapeutic portfolio for tremor, but several oral drugs, including long‐chain alcohols, T‐channel blockers, allosteric modulators of potassium channels, and of GABA‐A receptors, are currently being tested and hold promise. This review will discuss the key milestones in tremor research of the last 10 years, with a focus on the most common tremor syndromes, namely essential tremor, dystonic tremor, and Parkinsonian tremor.

At the time of writing, 10 years has passed since the article “Milestones in Tremor Research” by Rodger Elbe and Gunther Deuschl was published in *Movement Disorders*.^1^ Based on a review of the previous 25 years, they highlighted the advances in tremor research and eventually concluded that it was “time to refine the classification and diagnostic criteria for various tremor disorders, particularly Essential Tremor (ET) and dystonic tremor (DT)”.^1^ This paper will serve as a starting point for the current work, in which we discuss the key advances of the last decade in terms of clinical concepts, tremor measurement, pathophysiology, and treatment approaches with a focus on the most common tremor syndromes, namely ET, DT, and Parkinson's disease (PD) related tremor. Conversely, this review will not go into etiological considerations, owing to the lack of consistent findings especially with regard to ET. The interested readership is referred elsewhere for consideration of the diverse etiologies, including genetics, that might sustain the syndrome of ET^2^, ^3^, ^4^ as well as its heterogeneous post‐mortem findings.^5^, ^6^, ^7^, ^8^

## Clinical Concepts

In 2018, the Tremor Task Force of the International Parkinson's and Movement Disorders Society (IPMDS) published the new tremor classification,^9^ this probably being the most notable advance of the last decade. Mirroring the recent changes in the dystonia field, the main structure of the classification is based on two axes: clinical features (axis I) and etiology (axis II). The inspiring aim of the new classification is to facilitate deeper and detailed phenotyping of patients with tremor, given the failure of having identified robust pathophysiologic and etiologic correlates of any tremor syndromes, particularly of ET. In fact, decades of research on ET with its loosely defined boundaries have led to a lack of consensus about its epidemiology, defining clinical features and prognosis, neurophysiologic markers, and pathology.^10^ Therefore, the argument has been put forward that “ET may have more than one etiology and vice‐versa an etiology of ET could conceivably produce more than one clinical syndrome”.^9^ Accordingly, ET has been re‐conceptualized as a clinical syndrome (axis I), rather than a single disease entity, consisting of an isolated bi‐brachial action tremor of at least 3‐year duration.^9^ The 3‐year time frame is admittedly arbitrary, and the entity of “indeterminate tremor” was coined for those seemingly ET patients with a shorter disease duration.

Furthermore, the construct of “ET‐plus” was introduced for those patients fulfilling the criteria of ET, but also having either a rest tremor or additional “soft signs” that do not suffice to make an alternative diagnosis.^9^ Reclassification of formerly diagnosed ET patients according to the new criteria has evidenced that ET in absence of soft signs would be less common than ET‐plus.^11^

The construct of ET‐plus represents another notable departure from the former tremor classification and owes to the lack of consensus among the panel of experts on which of these additional (soft) signs were acceptable within the definition of ET.^4^ The most prominent criticism raised by some researchers against the construct of ET‐plus stands in the lack of pathological differences between ET and ET‐plus.^12^ However, this criticism misinterprets that: (1) for neither tremor syndrome is there a generally accepted underlying pathology; and (2) that both ET and ET‐plus are currently viewed as syndromes rather than single diseases.^13^ Nonetheless, the construct of ET‐plus has generated an enlightened debate about its validity,^12^, ^14^ with some researchers proposing that it could represent a later stage of ET based on the evidence that its defining characteristics vary as a function of disease duration.^15^ It should be noted, however, that the transition between syndromic allocations (ie, from ET to ET‐plus) can occur in the other direction (ie, from ET‐plus to ET because of the resolution of the soft signs).^16^ This example highlights one of the novelties of the current classification, that is, the possibility that one syndrome might “evolve” over time into another. Tracking transitions across different tremor syndromes will eventually clarify the controversial relationship between them, for example between ET and ET‐plus or between ET and PD,^17^ which is the reason why the idea of diseases with “antecedent ET” are described in the new classification.^9^

Importantly, some tremor syndromes that were loosely considered within the ET spectrum, including isolated head/voice tremor, task‐specific tremors and orthostatic tremor, have been now conceptualized as different and discreet entities,^9^ owing to accumulating evidence pointing to different pathophysiology.^18^, ^19^, ^20^

As discussed in more detail in the corresponding section, one possible caveat of the current classification is the lack of mention of pathophysiological mechanisms sustaining a particular syndrome. Using as an extreme example the recently described rare entity of “myogenic tremor”,^21^ the ET label might appear far‐fetched, given that the pacemaker has been construed to be located at the level of the myofilaments, although neurophysiologic studies have suggested tremor amplification by an additional central loop modulating the clinical phenomenology.^22^ Likewise, the border between ET and enhanced physiological tremor with a central component can be blurred and clinical differentiation between phenomenologically similar syndromes difficult in the absence of pathophysiological markers. Notwithstanding, the strict operational clinical criteria of the new classification will likely provide a rigorous framework for future translational research.

## Tremor Measurement

Transducers have been used in the study of tremor for more than 100 years, but the large infusion of smartphones, tablets, and smartwatches has fostered the development of specialized software especially in the last decade, which by using on‐board sensors, provide very precise linear measures of tremor as opposed to imprecise non‐linear measures obtained by clinical ratings.^23^ However, it should be noted that these recording and analysis procedures have not been strictly defined, so that the best protocol for tremor assessment has not yet been determined.^23^ A number of factors including anatomic placement of the transducer, selection of motor task versus recording during spontaneous unconstrained activities, duration of sampling, and methods of spectral analysis, will influence tremor measurements.^23^ Probably the hardest challenge to face relates to an inherent feature of tremor, namely within‐subject fluctuations. Tremor changes because of disease progression or treatment cannot be captured until they exceed this natural variability. This likely explains the failure in detecting tremor progression using sensor‐based measures rather than what would be obtained by clinical ratings alone.^24^ Nonetheless, detailed characterization of tremor features by means of sensors may be helpful in the differential diagnosis between tremor syndromes,^25^, ^26^ to predict therapeutic outcomes,^27^ and to adapt deep‐brain stimulation (DBS) paradigms to individual tremor physiology.^28^, ^29^ It is, therefore, expected that transducers will be increasingly used in both clinical and research settings, also because they enable more frequent and/or longer tremor assessments, and they can be used almost anywhere without clinician raters and/or might be exploited to substantiate clinical ratings.^23^

The advances in the instrumental measurement of tremor reflect on the possibility of developing novel treatment strategies based on tremor characteristics, which can be sensitively measured only by means of sensors, which is why they are discussed in this section. For example, the detailed exploration of tremor characteristics with transducers, namely tremor phase, might allow phase‐locked non‐invasive stimulation as a new tool for treatment. Therefore, both in‐phase and out‐of‐phase electrical muscle stimulation paradigms with pulse intensity over the motor threshold (ie, able to induce a muscle contraction) have been used with promising results.^30^, ^31^ The rationale is either to increase impedance at the tremulous joint or to generate counteracting forces in antagonist muscles that are opposite to those that generate tremor. Similarly, low‐level, afferent electrical stimulation tuned to the tremor frequency has been also shown to reduce tremor.^32^, ^33^ Finally, a recent work has developed a strategy to compute the instantaneous phase of tremor in ET and applied a phase‐locked cerebellar transcranial alternating current stimulation (tACS), further demonstrating that tremor amplitude reduction was attributable to a disruption of the cascade of coherent activities in the downstream loop.^34^

## Pathophysiology

One aspect that has been overlooked by the current classification of tremor, arguably because is out of its scope, is related to the pathophysiology. This let some authors to propose a pathophysiology‐guided axis III.^35^ In fact, different pathophysiological processes sustaining a similar syndrome are likely related to diverse etiologies. On the other hand, in a single disease multiple tremor types might be present as result of different pathophysiological processes. Both examples highlight how a pathophysiology‐guided axis III might fill the gap between clinical aspects (axis I) and etiology (axis II). This is particularly relevant to the relationship between dystonia and both the ET‐like tremors that some patients might present (the so‐called “tremor associated with dystonia” [TAWD]) as well as ET in general. Likewise, such pathophysiology‐guided axis might be useful in understanding the biological underpinnings of rarer forms of tremor, including task/position‐specific tremor and isolated head/voice tremor.^18^, ^19^

Expanding on, yet somehow diverging from the “olivary model” of ET,^36^ it is now accepted that the key system involved in the pathophysiology of many tremors is the cerebello‐thalamo‐cortical (CTC) circuit, with other inter‐connected areas including the basal ganglia,^37^ the supplemental motor area and other cortical areas,^38^, ^39^, ^40^ and the brainstem,^20^ also being affected and potentially explaining the clinical differences between tremor syndromes. Notably, evidence from imaging and well as electroencephalographic/magnetoencephalographic studies have revealed a dynamic entrainment of multiple nodes of this network,^41^, ^42^, ^43^, ^44^ which would lead to a rhythmic modulation of muscle activity becoming apparent as tremor. However, it remains unclear where the oscillations primarily originate within the CTC network with different, alternative hypotheses being proposed.

A body of work suggested an increased cerebellar drive in ET (cerebellar oscillator hypothesis).^39^, ^45^ This might be sustained by synaptic pruning deficits of climbing fiber to Purkinje cell synapses,^45^ which retrieves the inferior olive as a major spot of tremor generation.^36^ Moreover, alternative evidence has suggested a key mechanism in the disconnection of cerebellar output pathways (cerebellar decoupling hypothesis).^40^, ^46^ Notably, each of these abnormalities might not be present in all ET patients as suggested by studies comparing early‐ and late‐onset cases,^47^ sporadic and familial patients,^48^ or by indirect clinical evidence demonstrating different tremor characteristics across different activating conditions in ET,^49^ which would point to different pathophysiological mechanisms.^50^ Of note, most of these studies have been carried out before the new tremor classification was published and it is, therefore, unknown whether these concepts apply to both ET and ET‐plus.

Interestingly, the spectrum of tremor types occurring in dystonia syndromes might be also sustained by different mechanisms. Although some authors suggested a cerebellar involvement in both DT and TAWD,^37^, ^51^ others supported a prominent role of basal ganglia in DT^52^ or suggested differential pathophysiology between the two types of tremor, with DT being closer to non‐tremulous dystonia and TAWD to ET.^53^ Furthermore, evidence arising from pallidal single‐neuron recordings in cervical dystonia would support the notion that phenomenologically different tremors (ie, sinusoidal vs. jerky tremor) might have different pathophysiology,^54^ arguably independent from tremor distribution. Therefore, sinusoidal tremor (ie, “ET‐like”) would be driven by cerebellar alterations whereas jerky tremor by pallidal alterations.^54^

Somewhat similarly, it has been shown that different types of tremor occurring in PD might relate to different pathophysiologic mechanisms. Therefore, some PD patients exhibit a pure postural tremor, which is not re‐emergent, has a higher frequency than the rest component, and does not respond to levodopa, all features contrasting with the re‐emergent tremor that is highly correlated with resting tremor.^55^ Interestingly, such dopamine‐resistant tremor depends on an increased tremor‐related activity in the cerebellum, whereas patients with dopamine‐responsive tremor have increased thalamic ventralis intermediate nucleus (VIM)‐cortical activity.^56^, ^57^ These concepts have been summarized as the “dimmer‐switch” theory of PD tremor formulated by Rick Helmich and colleagues.^58^

**FIG. 1:**
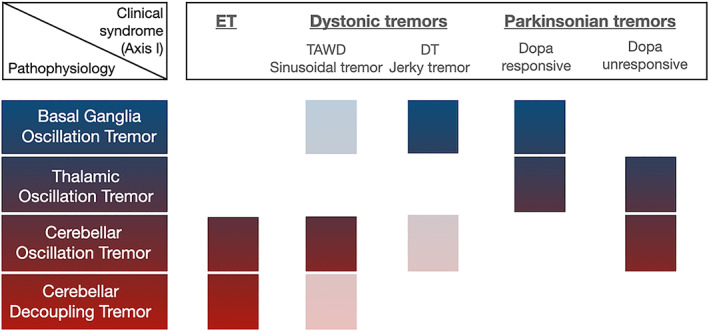
Suggested pathophysiological mechanisms sustaining essential tremor, dystonic tremors, and parkinsonian tremors (whitened boxes indicate a possible but minor contribution; redrawn from van der Stouwe et al).^35^ ET, essential tremor; TAWD, tremor associated with dystonia; DT, dystonic tremor.

In summary, significant advances in the last decade have highlighted that different pathophysiologic mechanisms, at least in terms of involved circuitry, might occur across and within different tremor syndromes (Fig. 1). It should be noted however, that the demonstration of specific neuro‐imaging and/or electrophysiological changes are permissible within axis I of the new tremor classification, according to which information from four main domains (ie, historical features, tremor characteristics, associated signs, and, indeed, findings from additional laboratory tests) should be gathered.^9^ This emphasizes the concept that the definition of a clinical syndrome by axis I should not be made solely on features that can be collected with the naked eye. Nonetheless, the current classification does not require any additional testing for the formal definition of the proposed tremor syndromes,^9^ not even the loading test for the distinction between mechanical‐reflex and central neurogenic tremors. On the contrary, some easy‐to‐collect neurophysiological measures (ie, the tremor stability index, which is a proxy of variability in the tremor period or frequency) have been suggested to accurately differentiate between different tremor syndromes, namely ET and PD‐related tremor.^59^

Hence, the identification of the pathophysiological underpinnings sustaining the clinical variability within and between different tremor syndromes and/or the recognition of physiological markers reflecting this variability might theoretically have therapeutic implications, as continued below.

## Treatment

There have not been significant advances in the commercialized pharmacological treatment of tremor, as appraised in the 2019 IPMDS evidence‐based review.^60^ Nonetheless, it is worth noting that, based on positive preclinical evidence, long‐chain alcohols including 1‐octanol and its metabolite octanoic acid (OA) have been recently tested in ET. Despite some evidence of efficacy, the actual viability of 1‐octanol therapy is limited by its pharmacological properties that require large volumes to be orally administered.^61^ Conversely, the first trial using OA demonstrated excellent safety as well as efficacy in secondary, but not primary, outcome measures of tremor amplitude.^61^ It is expected that OA will be tested in additional phase‐2 trials, perhaps targeting different tremor syndromes. More importantly, for the first time in history a number of companies have anti‐tremor drugs in the pipeline. These are T‐channel blocking agents (CX‐8998, PRAX‐944), an allosteric modulator of small conductance calcium‐activated potassium channels (CAD‐1883), and allosteric modulators of GABA‐A receptors (brexanolone, SAGE 217 and 234).^62^

Surgical treatment of tremor has become standard of care in many countries and is now an integral part of the anti‐tremor portfolio. New technologies are being implemented for VIM DBS in particular. These include the use of directional stimulation^63^ and/or short pulse width,^64^ technologies able to expand the therapeutic window of stimulation, also making safer the use of bilateral DBS in terms of balance and other ataxia side effects of stimulation.^65^ Adaptive DBS (aDBS) of VIM is only investigational at the moment but preliminary reports on two subjects also implanted with electrocorticography strips over the hand portion of M1 to close the loop, have provided initial evidence of its feasibility.^66^, ^67^ Building on previous pathophysiological studies,^29^, ^43^ future aDBS approaches will use thalamic recordings to close the loop thereby stimulating the oscillatory activity of these neurons only during certain phases. The possibility of thalamic recording in tremor patients is already feasible with recently commercialized DBS devices.^68^ How these new technologies will reduce the still unclear phenomenon of DBS “habituation” (or “tolerance”) is uncertain although aDBS seems to be the most promising approach in this regard.^69^ Nevertheless, long‐term prospective studies of patients treated with VIM DBS are now available and overall indicate a reasonably sustained benefit in most ET patients.^70^

MRI‐guided focused ultrasound (MRgFUS) thalamotomy has been the most interesting advance in the surgical treatment of tremor during the past 10 years. MRgFUS uses over a thousand transducers to focus ultrasound beams on a precise brain location, therefore, creating a coagulative lesion, a thalamotomy in the case of tremor.^71^ This therapy is now approved for ET in many countries, as a number of prospective studies—including a randomized sham‐controlled blind trial—have been conducted.^72^ MRgFUS thalamotomy has been conducted also in other tremor syndromes, including DT and PD‐related tremor.^73^ Tremor outcome in the short term is comparable to VIM DBS although a decay of benefit, requiring repeated treatment, has been consistently reported.^74^ Safety profile is satisfactory and the absence of craniectomy and general anesthesia makes elderly patients suitable candidates.^75^ More recently, staged bilateral MRgFUS thalamotomy has been shown to be both effective and safe in ET patients.^76^

Moreover, the advances in neuroimaging have allowed a more precise targeting for all surgical therapies of tremor, particularly through the direct visualization of the dento‐rubro‐thalamic tract.^77^ Imaging has also allowed the understanding of the “sweet spots” of VIM DBS^78^ as well as MRgFUS.^79^, ^80^

Last, a novel venue of research has recently explored the possibility of suppressing tremor by means of non‐invasive stimulation approaches. Therefore, expanding on the results of Schreglmann and colleagues^34^ who successfully applied a phase‐locked cerebellar tACS in ET, Nieuwhof et al^81^ adopted an identical approach to dystonic tremors demonstrating that phase‐locked cerebellar tACS modulated tremor amplitude solely in patients with sinusoidal tremor, but not in patients with jerky (irregular) tremor. This reflects on the concept that the cerebellum plays a causal role in the generation of sinusoidal dystonic tremor syndromes (Fig. 1) and further opens the question of whether differential targets of DBS (ie, cerebellar relay vs pallidal relay thalamic nuclei) should be tailored according to the specific circuitry involved in tremor generation.^49^, ^82^

## Conclusions

The past decade has generated major advances in our knowledge about tremor, paving the way toward the unmet needs in the field, which are the discovery of the different etiologies that cause tremor and the development of pathophysiology‐driven treatments. The next steps on the roadmap to attain these objectives will keep us busy for the next decade.

## Author Roles

(1) Conception and design of the study, or acquisition of data, or analysis and interpretation of data; (2) Drafting the article or revising it critically for important intellectual content; (3) Final approval of the version to be submitted.

R.E.: 1, 2, 3

A.F.: 2, 3

P.B.:2,3

K.P.B.: 2, 3

## Disclosures

### Ethical Compliance Statement

The authors confirm that the approval of an institutional review board and patient consent was not required for this work. We confirm that we have read the Journal's position on issues involved in ethical publication and affirm that this work is consistent with those guidelines.

### Funding Sources and Conflicts of Interest

No specific funding was received for this work. The authors declare that there are no conflicts of interest relevant to this work.

### Financial Disclosures for the Previous 12 Months

R.E. receives royalties from publication of Case Studies in Movement Disorders—Common and Uncommon Presentations (Cambridge University Press, 2017) and of Paroxysmal Movement Disorders (Springer, 2020). He has received consultancies from Sanofi and honoraria for speaking from the International Parkinson's Disease and Movement Disorders Society.

P.B. received consultancies as a member of the advisory board for Zambon, Lundbeck, UCB, Chiesi, Abbvie, and Acorda.

K.P.B. has received grant support from EU Horizon 2020. He receives royalties from publication of the Oxford Specialist Handbook Parkinson's Disease and Other Movement Disorders (Oxford University Press, 2008), of Marsden's Book of Movement Disorders (Oxford University Press, 2012), and of Case Studies in Movement Disorders—Common and uncommon presentations (Cambridge University Press, 2017). He has received honoraria/personal compensation for participating as consultant/scientific board member from Ipsen, Allergan, and honoraria for speaking at meetings from Allergan, Ipsen, and the International Parkinson's Disease and Movement Disorders Society.

A.F. has received consultancies from Apple, Abbvie, Abbott, Medtronic, Boston Scientific, Sunovion, Chiesi farmaceutici, UCB, Ipsen, and Rune Labs; he serves on the Advisory Boards of Abbvie, Boston Scientific, Ceregate, Gondola, Inbrain, and Ipsen; he has received honoraria from American Academy of Neurology, Abbott, Abbvie, Medtronic, Boston Scientific, Sunovion, Chiesi farmaceutici, UCB, Ipsen, Paladin Lab, and Movement Disorders Society; he has received royalties from Springer; he has received grants from the University of Toronto, Abbvie, Medtronic, Boston Scientific, MSA Coalition, and McLaughlin Centre.
